# Pneumonia

**Published:** 1896-01

**Authors:** C. D. Hurt

**Affiliations:** Atlanta, Ga.


### ATLANTA
### Medical and Surgical Journal.

Vol. XII. JANUARY, 1896. No. 11.

LUTHER B. GRANDY, M.D., MANAGING EDITOR.

M. B. HUTCHINS, M.D., BUSINESS MANAGER.

COLLABORATORS:
H. V. M. MILLER, M.D., LL.D., VIRGIL O. HARDON, M.D., FLOYD W. McRAE, M.D., AND DUNBAR ROY, M.D.

### *ORIGINAL COMMUNICATIONS.*

### PNEUMONIA.

By C. D. HURT, M.D.,

Atlanta, Ga.

Pneumonitis is an inflammation of the lungs. Some authors hold that it is infectious and caused by pneumococci, which find their way into lung tissue and excite inflammation, while others hold that it is the direct application, either through the respiratory passages, or by undue exposure of the body to cold and damp atmosphere.

Pneumonia appears in all climates and at all seasons; in both sexes, at all ages. Among adults, males are more frequently affected than females. It prevails most in winter season and early spring. Pneumonia is more frequent among those who are exposed to hardships, to inclement weather, to irregular habits of diet and exercise. A general lowering of the physical and mental forces renders one more susceptible. Those who are alcoholics habitually or intemparately are peculiarly liable to attacks.

While climate exerts but little influence, certain sections show a greater prevalence. It is said that in the United States it prevails to a greater extent in the Southern portion, and is accounted for in the sudden changes from heat to cold. In the Northern States the thermal range in winter is more uniformly low, and the clothing and dwellings afford better protection.

Sudden chilling of the body, either by cold air or getting the clothing saturated with water, was at one time regarded as the sole cause; but during the last two decades much has been argued in favor of pneumococci producing it independent of cold. It is further argued that pneumonia is epidemic. Traumatic pneumonia is denied by none, nor is it claimed that pneumococci in the system are responsible for its development under such conditions. If traumatism alone is responsible for pneumonia under such conditions, it remains for us to determine what influences may operate like traumatism and independent of pneumococci as a cause. Cold is capable of producing local disturbances, which in effect simulate traumatism. Traumatism results also from the application of heat, whether it be the direct application of a hot poker, boiling water, or over heated atmosphere. Cold is the antithesis of heat, and why is it not capable of producing traumatism, and why is it not called traumatism ? With all the efforts which have been made theoretically to attribute pleurisy to other causes than cold, the most zealous advocates still admit that it does follow with striking rapidity a certain wetting or chilling of the body. It must not be forgotten that cold air is frequently made to displace warm air in the respiratory act with such suddenness as to chill lung tissue and establish irritation which not infrequently develops inflammation (pneumonia). If traumatism produces pneumonia independent of pneumococci, or a soil previously prepared for their cultivation, and if, as has been shown, heat and cold may be so applied as to produce traumatism, who can gainsay the statement that cold (or heat for that matter) is the cause of pneumonia? We know that cold produces coryza, conjunctivitis, arthritis, muscular inflammation, and pleurisy. Then when the lung is exposed to cold respired, as well as external air, why should we deny the cause? Is it because the pneumococcus has been found in the sputa during the existence of pneumonia? These are admitted to exist in the saliva of persons who have not had pneumonia, and may also be found for months after one has recovered from the disease.

The important question is, are we to ascribe acute pneumonia to pneumococci which lie in the system dormant and harmless until as by force of will, they shall attack the vital organ and terminate one's existence; or shall we say that these microbes wait for some power to convert lung tissue into a proper culture medium in which they shall rise in their strength to accomplish their work of destruction? Or shall we say that traumatism produces pneumonia, and that cold properly applied produces traumatism?

Much depends upon a proper answer to this question, and we should settle it in our minds before we are properly licensed to treat the disease. One author says, on the view just stated, pneumonia may be regarded as a local disease, produced by inhalation of the diplococcus. Another says it is a local disease produced by cold (traumatism) or other local injuries. Klemperer brothers, of Leyden's clinic, have been experimenting, and claim that a person may not only be made immune, but can be cured of pneumonia by the injection of immunizing fluid obtained from bouillon cultures. Many authors of recent date and modern theories hold that pneumonia is a self-limited disease. This idea is in keeping with Klemperer brothers' experiment, as it claims that the pneumonic patient immunizes himself after a few days, the temperature falls, the crisis passes, and the patient goes on to recovery. This theory might hold if all pneumonia patients recovered, or the death-rate under this theory, adopted in practice, was not so largely increased.

Pathologists are still all agreed that pneumonia embraces three stages or conditions of the lung, viz.: Engorgement, solidification, and hepatization. In that of engorgement the lung tissue is filled with blood and serum; in the stage of solidification or red hepatization the tissue becomes firmly matted with fibrinous mass. In gray hepatization the air cells are filled with leucocytes, and the red corpuscles have disappeared.

These conditions primarily affecting lung tissues, begin to disturb the equilibrilum of the general circulation, the heart becomes weary of being overloaded in the right side with obstructed venous blood, and the left heart clamors for its normal stimulant, fresh, oxygenated blood. One of the dangers most dreaded in pneumonia is heart failure. This heart failure may result early in the history of a case, that is in its congestive stage. As congestion comes on diminished oxidation of blood takes place, and for want of proper nourishment and stimulant, the vagi becomes less active and less capable of keeping up the normal heart action. At the same time the overloaded right heart tires and muscular power is lessened.

The diagnosis of pneumonia is so easy and unmistakable, that it is unnecessary in the purpose of this article to speak of it. But on account of the varying opinions as to cause, we take up the question of treatment. Pneumonia is claimed by some to be a self-limited disease, to run its course uninfluenced by medicine.

Osler says, "it can neither be aborted, or cut short in its course by any known means at our command. Even under the most unfavorable circumstances it will terminate abruptly and naturally, without a dose of medicine having been administered." That mild cases can, and do recover without medicine, is not denied. The same is true of all zymotic diseases. This fact does not argue self-limitation resulting in recovery. A large per cent. of pneumonia cases prove fatal, and under the modern treatment a larger death-rate is recorded. In other the expectant treatment, the let alone treatment, and the treatment based upon the theory that it is a constitutional disease, have all failed to lessen and tended to increase the death-rate. Are we not led into the delusion because of our extended and yet incomplete knowledge of the germ theory? If pneumococci are present (as claimed by Netter) in twenty per cent. of healthy persons, why should we not expect to find them in certain unhealthy or diseased persons? Why, if in twenty per cent. of healthy persons do not statistics show a greater per cent. of pneumonia cases? The only rational conclusion to be drawn from such a statement is that the animal system must be brought into the condition of a proper culture medium before the pneumococcus is offensive and harmful. What influences may bring the system into this proper culture medium? We have pneumonia from disintegration of lung tissue under advanced tuberculosis. We have it from traumatism, and it begins frequently under such injuries within a few hours. Traumatism may consist in a severe concussion of the lung from a fall or blow; from the irritation produced by the rugged ends of one or more fractured ribs; from incised or perforated wounds of lung tissues; from inhalation of strong acids or other irritating substances; from disturbing influences, either heat or cold, which cause primarily passive or active congestion of blood in the capillaries of the lungs, and as a sequel, inflammation. Whether pneumonia is from local or constitutional causes, whether it is produced by pneumococcus or is independent of it, pathologists all agree as to the morbid condition of lung tissue, and the danger of heart failure when pneumonia runs its full course, but therapeutists are widely at variance as to the best modes of treatment. The condition of the lung in the first stage of pneumonia is that of engorgement. If resolution can be secured from this stage, the fluid blood which has here been detained is allowed to speed on its way unchanged, and with only temporary disturbance to other organs and functions, and the patient is soon restored.

The second stage of pneumonia is that of solidification; that is, the blood has deposited more or less of its fibrin in shreds and masses into lung tissue, the serum having been forced into separate apartments, the blood which should be propelled by the right heart, meets with obstruction at this fibrin, and the pulmonary arteries and right heart being over-distended, are likewise overtaxed and the natural tendency is to heart failure. At the same time the left fails to receive its normal supply of healthy oxygenized blood, nutrition is lessened and the escape of thrombi from the lung tissue along the pulmonary veins into the left heart is liable to occur.

In the third stage, that of gray hepatization, the air cells are filled with lucocytes, the lung tissue becomes more friable, and the case is nearing the border line of suppuration. This condition is a higher degree of morbid anatomy, and produces greater risk to the patient, and greater tax to recovery from it. The majority of cases have more or less pleurisy as a complication, and the risk of its sequelæ.

Now, as to the treatment in these different stages. Why not endeavor to at once begin the work of resolution, or remove the morbid anatomy without allowing the disease to run through its consecutive courses.

Death frequently occurs in four or five days, and the advocates of the theory of self limitation, the self immunizing theory, and of the let alone theory, are robbed of their clients.

In the early stage, if the patient is stout, vigorous, and of full habit, a mercurial cathartic should be given. If the patient is asthenic a mild laxative to empty the bowels is sufficient.

Anodynes to relieve pain may be repeated in small doses, *pro re nata.* Antipyretics, if judiciously used to control fever, add to the comfort of the patient. Quinine in large doses is objectionable, but is tolerated with benefit in small doses. Of the coal tar derivatives, phenacetine is safest and most pleasant in effect. Veratum viride, much abused in former years, still has its advocates, and is a valuable remedy in persons of full habit. Venesection in early stage, if the pulse is full and dyspnea great, is of material benefit. The prejudice against bleeding is being rapidly overcome, and already our eastern confreres are returning to its use. It relieves the tension upon the blood vessels and dissipates the engorgement which is found in the lungs. Local extraction of blood vessels from the chest wall by cupping or leeching is advocated by some, and is not without temporary relief. These local means are more admissible in asthenic patients with feeble constitutions, whose strength must be preserved. But in stout and vigorous patients in the first stage, where the engorgement and dyspnea are not relieved by milder treatment, vesication with cantharides covering an area more extensive than the portion of inflamed lung, is attended with immediate relief, and subsequent arrest of inflammation. Disappointment in the use of blisters has resulted from timidity in not covering enough surface. The vesication should be shallow, but complete in its extent, and the after-dressing should be warm poultices of starch or flax seed meal.

The application of cotton batting over the chest serves a good purpose, and applied early and continuously, may obviate the need of a blister. The silk jacket is, alone, worthless and serves only to console the medical attendant in his waste of valuable time.

In feeble constitutions, with weak heart and quick pulse, stimulants, carbonate of ammonia and whisky are demanded.

